# Supplementary material for: Mutations in PCYT2 disrupt etherlipid biosynthesis and cause a complex hereditary spastic paraplegia
Source: Brain. 2019 Oct 22;142(11):3382–97. doi: 10.1093/brain/awz291 (PMC6821184; doi:10.1093/brain/awz291)
Supplement: awz291_Supplementary_Data [file awz291_supplementary_data.zip › awz291-Suppl_data/Supplementary_Data4.zip!/Supplementary information 4 - plasma lipidomics.zip!/Plasma lipidomics/Control vs Patient3a/Statistical plots and data/PCA.pdf]

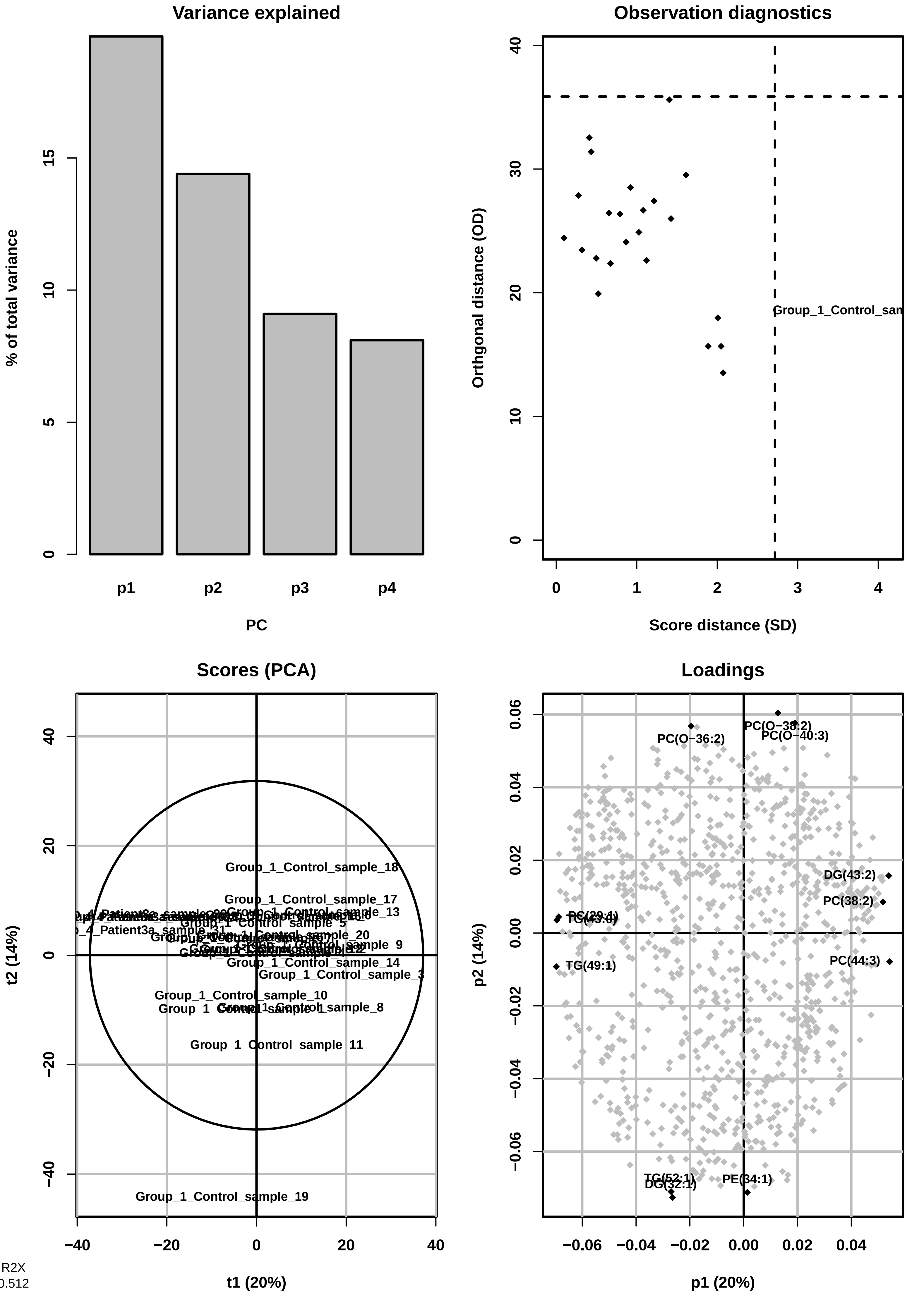

Variance explained
% of total variance
0
5
10
15
p1
p2
p3
p4
PC
Observation diagnostics
Orthgonal distance (OD)
Score distance (SD)
Group_1_Control_sam
Scores (PCA)
t1 (20%)
t2 (14%)
Group_1_Control_sample_18
Group_1_Control_sample_17
Group_1_Control_sample_13
Group_1_Control_sample_5
Group_1_Control_sample_20
Group_1_Control_sample_9
Group_1_Control_sample_14
Group_1_Control_sample_3
Group_1_Control_sample_10
Group_1_Control_sample_8
Group_1_Control_sample_11
Group_1_Control_sample_19
Loadings
p1 (20%)
p2 (14%)
PC(O−38:2)
PC(O−36:2)
PC(O−40:3)
DG(43:2)
PC(38:2)
PC(29:1)
TG(43:0)
TG(49:1)
PC(44:3)
TG(52:1)
DG(32:1)
PE(34:1)
R2X
